# The Reporting Quality of Acupuncture-Related Infections in Korean Literature: A Systematic Review of Case Studies

**DOI:** 10.1155/2015/273409

**Published:** 2015-11-03

**Authors:** Tae-Hun Kim, Jung Won Kang, Wan-Soo Park

**Affiliations:** ^1^Korean Medicine Clinical Trial Center, Korean Medicine Hospital, Kyung Hee University, Seoul 02447, Republic of Korea; ^2^Department of Acupuncture & Moxibustion, College of Korean Medicine, Kyung Hee University, Seoul 02447, Republic of Korea; ^3^College of Korean Medicine, Gachon University, Seongnam 13120, Republic of Korea

## Abstract

*Objective*. Acupuncture is generally accepted as a safe intervention when it is administered in appropriate clinical setting by well-educated and experienced practitioners. In this study, we reviewed observational studies on adverse events (AEs) or complications relevant to acupuncture practice in Korean literature for assessing their reporting quality and suggested recommendations for future ones on acupuncture-related infections. *Method*. Electronic databases including Medline, Embase, Cochrane library, Korean studies Information Service System, DBpia, National Digital Science Library, and Korean National Assembly Library were searched until May 2015. Combination of keywords including “acupuncture” and “infection” were used for searching databases. *Result*. A total of 23 studies from 2,739 literature articles were identified from electronic database searching until May 2015. From this review, we found that most case studies did not report enough information for judging causality between acupuncture and the AEs (or complications) as well as appropriateness of the acupuncture practice. In addition, acupuncture experts rarely participated in the reporting of these AEs (or complications). *Conclusion*. Based on these limitations, we suggest a tentative recommendation for future case studies on acupuncture-related infection. We hope that this recommendation would contribute to the improvement of the reporting quality of acupuncture-related AEs (or complications) in the future.

## 1. Introduction

Acupuncture is a considerably safe intervention when it is administered in an appropriate clinical setting by well-educated and experienced practitioners. According to a large-scaled prospective survey result, only self-limited minor adverse events (AEs) (e.g., bleeding and needling pain) occurred in few cases with an incidence rate of 671/10,000 consultations [1]. Moreover, most of the severe complications of acupuncture are mainly related to the improper practice settings and inexperience of half-fledged practitioners. In particular, acupuncture-related infections have been reported to be closely related to the poor usage of acupuncture and disinfection procedures [2].

By the way, acupuncture-related AEs have been reported continuously, and the number of AE cases has not decreased even until recently [3]. The most serious problem among these AE cases was that essential information for judging the appropriateness of the acupuncture practice was not reported clearly [3, 4]. In this sense, case studies on acupuncture-related AEs are not regarded to be adequately informative to reduce errors and to enhance safety in future acupuncture practices and only lead to exaggeration of acupuncture-related harm among the general public. For predicting future AEs and promoting prevention measures in avoidable AE cases, it is important to identify whether the AEs and complications in the past studies were caused by negligence in practice or adverse reaction to acupuncture, which is not possible to determine retrospectively.

Reporting guidelines have been developed for transparent and easy reporting of clinical trials [5] and cases studies [6]. Regarding AEs, the Consolidated Standards of Reporting Trials' (CONSORT) AE reporting guideline is available for clinical trials [7]. Guideline for acupuncture-related AE reporting was suggested earlier [8], but it has not been adopted frequently in the case studies since its publication. There are both similarities and differences between AEs related to acupuncture practice and those of drug-related AEs in some aspects; therefore, the guideline for acupuncture-related AEs should include common components such as description of patient's demographic data, medical history, and risk factors for, detailed condition of, and clinical outcomes of the current AEs [9] as well as acupuncture-specific aspects such as details of acupuncture intervention (e.g., needling points, needling depth, practitioner's types and experience level, and acupuncture materials used) [8]. In addition, evaluation of the appropriateness of acupuncture practice and the correlation between acupuncture and AEs, which is a key for improving quality of future practice, need to be reported.

Acupuncture-related infection is one of the commonly reported AEs of acupuncture. Compared with other types of AEs, it shows comparatively low incidence [2], but it is considered to be one of the major issues for good acupuncture practice, because infection often introduces considerable harm to patient's safety [3, 4]. Considering this situation, transparent and rigorous reporting on acupuncture-related infection is necessary for acupuncture practitioners as well as medical consumers.

In this sense, the purpose of this study was to assess the status of the reporting quality of case studies on acupuncture-related AEs or complications, especially focusing on acupuncture-related infections in the Korean literature, and to suggest tentative reporting guidelines for future case reports.

## 2. Methods

This is a systematic review of the observational studies on acupuncture-related infections. In this review, we defined AEs and complications differently as follows: AEs and complications are common unexpected signs or symptoms related to acupuncture treatment, but AEs occur when acupuncture procedure is appropriate while complications are caused by the practitioner's negligence [10]. Distinction between these two concepts is crucial in the reporting of acupuncture-related infections, because complications are more relevant to individual practitioner's skill level and compliance with clinical guideline for safe practice including clean needle technique, whereas AEs are actually related to acupuncture itself.

Electronic databases including Medline, Embase, Cochrane library, Korean studies Information Service System (KISS), DBpia, National Digital Science Library (NDSL), and Korean National Assembly Library were searched until May 2015. Combination of keywords including “acupuncture” and “infection” was used for developing search strategies based on the characteristics and structures of the individual databases.

The search strategy for Medline was as follows: acupuncture AND (infection OR hepatitis OR HIV OR (auricular chondritis) OR endocarditis OR meningitis OR (spinal infection) OR septicaemia OR (necrotizing fasciitis and toxic shock) OR (septic arthritis) OR abscess OR skin OR herpes).

For this review, we only included case studies or series that reported infectious conditions related to acupuncture in Korean literature. Acupuncture covers a broad range of nondrug interventions including moxibustion in traditional East Asian medicine (TEAM) [11], but we only included the classic types of acupuncture (i.e., needle insertion into the skin) in this review. However, if other interventions including moxibustion, cupping, and blood-letting were added to classic acupuncture and it was impossible to define which intervention might be first cause of the AEs (or complications), we included them the study.

To assess the reporting quality of the individual studies, the following data were analyzed based on the literature: (1) data related to patient information: patient characteristics, disease or symptoms for which acupuncture treatment was performed, risk factors for AEs or complications, underlying diseases, detailed features of AEs or complications, final clinical outcomes and (2) data related to AE: occurrence time after acupuncture, laboratory or pathological findings, other possible causes, and spatial relationship between needling site and affected lesion. In addition, a description of acupuncture treatment including practitioner's type, needling site, depth of insertion, needle type, stimulation method, and acupuncture settings (i.e., where the acupuncture was practiced and disinfection procedure) was analyzed. Each item was judged as “well documented (WD)” when all the related information was reported appropriately in the literature, as “documented but not enough for the judgment (DE)” when information was suggested but not enough to clearly describe the patient's presentation and as “not documented (ND)” when no information was available in the literature.

Causality between the acupuncture and AEs (or complications) and appropriateness of acupuncture practice was evaluated subsequently. Causality was assessed according to the modified WHO-UMC causality assessment criteria: “Certain” when plausible time relationship between the event and acupuncture was observed in the literature without possible cause of other treatments or underlying diseases for AEs (or complications), “Probable” when reasonable time relationship was observed and AEs (or complications) are unlikely to be explained by other causes, “Possible” when reasonable time relationship was observed and AEs (or complications) were possibly explained by other causes, “Unlikely” when improbable time relationship was observed with other plausible causes, “Conditional” when more data from current undergoing examination was necessary for the evaluation, and “Unassessable” when information was insufficient for judgment [12]. The appropriateness of acupuncture was assessed based on the information of acupuncture practice in the literature: “Appropriate” when all the acupuncture procedure could not be a probable cause of the AEs (or complications), “Inappropriate” when any of the procedures might be the possible cause of the AEs (or complications), and “Unclear” when there was not enough information for the judgment of acupuncture procedure.

Data extraction and appraisal of causality and acupuncture appropriateness were conducted by two authors (Tae-Hun Kim and Jung Won Kang) independently. If there was any discordance between the two authors, which could not be solved by discussion, a third author (Wan-Soo Park) arbitrated them.

## 3. Results

**Figure 1:**
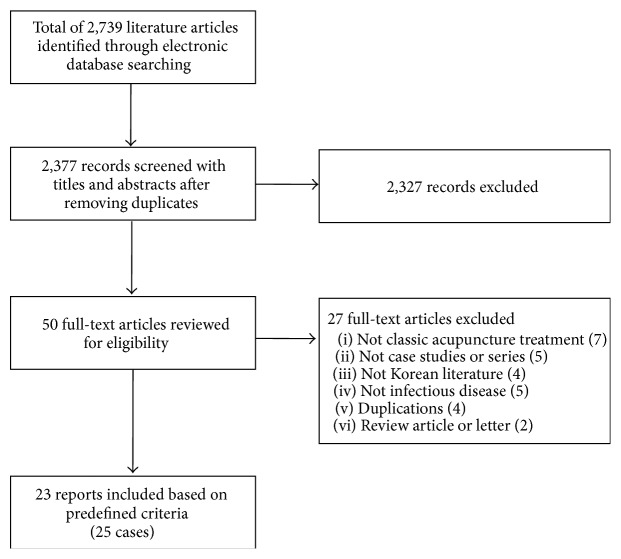
Study flow chart.

From the electronic database search, a total of 2,739 records were identified and 50 hard copies were reviewed for eligibility. Among them, 23 reports (25 cases) were included in this review (Figure 1). Revealed infectious agents were* Streptococcus* species in four studies [13–16],* Staphylococcus* species in four studies [17–20],* Mycobacterium* species in four studies [21–24],* Escherichia coli* in two studies [19, 25], and unidentified ones in three studies [26–28]. In addition, other infection agents were* Actinomyces* [29],* Bifidobacterium longum* [30],* Gemella morbillorum* [31], Herpes simplex virus [32],* Klebsiella pneumonia* [33],* Vibrio cholera* [34],* Serratia liquefaciens* complex [17], and* Spirochaete* [35]. The infectious diseases were skin infection in five studies [21–24, 32], sepsis in four studies [18, 25, 30, 34], fasciitis in three studies [15–17], psoas abscess in three studies [14, 25, 27], epidural abscess [20, 28] or inflammatory granuloma [26] in three studies, and others including abdominal actinomycosis [29], liver abscess [13], mediastinitis [31], necrotizing aortitis [19], pericardial abscess [18], spondylitis [25], retroperitoneal abscess [33], and syphilis [35]. Among 23 studies, only one study [28] consulted an acupuncture specialist who could analyze the AE (or complication) cases and judge the appropriateness of acupuncture practice as well as causality between the acupuncture and AEs (or complications) [28] (Table 1).

### 3.1. The Reporting Quality of the Information on the Patient and AEs (or Complications) Related to Acupuncture Practice

In most studies, the information on the patient's general characteristics, detailed features of AEs (or complications), and final outcomes of the AEs (or complications) were described very well. However, important patient's information including preceding conditions or reasons for seeking acupuncture (percentage of inappropriately reported studies, 22%) [19–21, 23, 33] and predisposing risk factors to which relevant AEs (or complications) might be attributable (70%) [17–26, 28–33] were not reported appropriately in most studies. Essential items for judging causality including other possible causes of AEs (or complications) (65%) [15, 16, 18–23, 25–27, 30, 32–34] as well as explanation of the association between needling site and the affected lesion (87%) [13–27, 30, 31, 33–35] were not suggested at all or insufficiently in most studies (Table 1). In this sense, all the included studies insisted that strong association between acupuncture treatment and the event existed, but causality of the acupuncture-related AEs (or complications) could not be concluded with the information provided in most studies (Table 2).

### 3.2. Reporting Quality of the Information on Acupuncture Practice

Although all the AEs (or complications) cases were asserted to be related to acupuncture, information on acupuncture and acupuncture practice were not reported adequately in most studies. Information on acupuncture practice closely relevant to acupuncture-related complications including practitioner's type (87%) [14–22, 24–27, 29–35], settings for acupuncture practice (74%) [14, 17–27, 29, 30, 32–34], needle types (100%) [13–35], usage of disposable, sterile needles (100%) [13–35], and features of acupuncture practice including needling site (96%) [13–27, 29–35], depth of insertion (100%) [13–35], stimulation method for acupuncture (100%) [13–35], and whether disinfection procedure was implemented properly (100%) [13–35] and was not described in most studies. So, appropriateness of acupuncture practice could not be appraised in all studies except for one [23] (Table 3).

## 4. Discussion

From this systematic review on the acupuncture-related infection case reports in Korean literature, we found that essential information for judging causality and appropriateness of acupuncture practice was not reported adequately. In particular, preceding risk factors, other possible causes, and spatial association between acupuncture needling and the affected lesion, which are necessary information when deciding causality, were not reported sufficiently. In this sense, we could not find concrete evidence in each report for the judgment of the causality between acupuncture practice and occurrence of the AEs (or complications) except for the time-order relationship. Description of the acupuncture procedure could not confirm the appropriateness of the acupuncture practice because it was insufficient and inadequate. Acupuncture specialists with expertise in these cases were not involved in the reporting process. These factors negatively affect the reporting quality of acupuncture-related AEs (or complications).

This review has several specific points. First, we assessed individual components in the case reports to identify possible errors related to the practice in these studies. When assessing the reporting quality of case reports on acupuncture-related infections, appropriateness of acupuncture practice should be evaluated. In this review, only one case had a complication that was definitely introduced by a wrong practice of acupuncture [23] and in other cases it could not be conclusively established whether the events were AEs or complications because the description was improper and deficient. However, previous systematic reviews on acupuncture-related infections only focused on the type and frequency of AEs but did not pay much attention to the appropriateness of acupuncture [4, 36]. Misconduct or errors during acupuncture can affect clinical outcomes; therefore, the assessment of appropriateness can give insight into future safe practice for acupuncture. Second, we evaluated causality rigorously based on the original case reports. To ensure transparency, two different authors whose specialty is acupuncture assessed causality individually and discussed the results. From this review, we found that most studies did not assess causality between acupuncture and AEs (or complications) appropriately, so case reports, although abundant, have limitations in that they do not help warn practitioners against the risks of acupuncture and are not informative enough to improve acupuncture practice. Causality of acupuncture-related AEs (or complications) should be assessed transparently and fairly based on the time association between the practice and the event, pathological mechanism of AEs (or complications), and exclusion of other potential causes [12]. In addition, acupuncture-related AEs (or complications) are different from drug-related AEs in that discontinuation and readministration of acupuncture hardly affect the clinical outcomes unlike with drugs [12]. Judging the causality between acupuncture and AEs (or complications), these factors need to be analyzed appropriately.

This study has limitations as well. First, we only assessed Korean literature, which reflects the reality of clinical practice in Korean context. Two different expert occupations in health-care sector, conventional medical doctors and Korean Medicine doctors, are in charge of national health with equal legal position, and competition among them is intensifying. Considering this conflicting situation, reporting of AEs (or complications) related to acupuncture might be overexaggerated (even with malicious intention) and not based on sound scientific evidence. Another explanation for this status could be that acupuncture specialists hardly participated in the case reporting of the included studies. The purpose of this study was to evaluate the quality of Korean literature, so this limitation is inevitable. Second, we only assessed acupuncture-related infections in this study. Apart from infections, other frequently reported acupuncture-related conditions such as traumatic injuries including pneumothorax and nerve injury need to be assessed in similar manner. We only assessed a small portion of acupuncture-related AEs (or complications), and the same issues on the reporting quality can be adapted to other types of AEs (or complications). These two limitations will be covered through further successive reviews in the future.

Based on the current review results, we now suggest a recommendation for reporting cases of acupuncture-related infections (Table 4). This recommendation includes items on patient's condition and AEs (or complications) in detail, which are necessary to establish the causality between acupuncture and the event and to provide information for judging appropriateness of acupuncture practice. Compared with a previously published reporting guideline for acupuncture-related AEs [8], this recommendation includes detailed information which is necessary for the judgment of causality between acupuncture and AEs (or complications) such as time and spatial relationship between acupuncture and AEs as well as the assessment of the other possible causes of AEs and appropriateness of acupuncture practice. In addition to this, we recommend that acupuncture specialists join as one of the authors for the AE reporting which is important for scientific and transparent reporting and lessons for future safe practice of acupuncture practice from the AE accident, which can be suggested as a clinical implication of the report. We hope that this tentative recommendation can be useful for reporting future cases of acupuncture-related infections in a clear and informative way as well as developing more advanced recommendation based on the future multidisciplined, mixed-method researches.

## Figures and Tables

**Table 1:** Reporting status on the information of patients and adverse events (AEs) or complications related to acupuncture practice in the included studies.

Study ID	Infection agents (numbers of the cases)	Final diagnosis	Inclusion of acupuncture specialist among the authors^*∗*^	Patient's information					AEs (or complications) information			
				Patient's characteristics	Preceding conditions or reasons for seeking acupuncture	Description on the risk factors for AEs (or complications)	Features of AEs (or complications)	Clinical outcome (follow-up)	Time relation between acupuncture and AEs (or complications)	Laboratory or pathological findings	Consideration of the other possible causes of AEs (or complications)	Explanation on the association between needling site and affected lesion
Bang and Lim 2006 [25]	*Escherichia coli* (1)	Psoas abscesses; epidural abscess; infectious spondylitis; sepsis	None	WD	WD	ND	WD	WD	WD	WD	ND	DE

Cho et al. 2003 [33]	*Klebsiella pneumonia* (1)	Retroperitoneal abscess	None	WD	DE	DE	WD	WD	WD	WD	DE	ND

Cho et al. 2010 [21]	*Mycobacterium abscessus* (1)	Mycobacterium abscessus cutaneous infection	None	WD	ND	DE	WD	WD	DE	WD	DE	ND

Cho et al. 2015 [31]	*Gemella morbillorum* (1)	Mediastinitis, osteomyelitis	None	WD	WD	DE	WD	WD	WD	WD	WD	ND

Choi et al. 2013 [13]	*Streptococcus intermedius* (1)	Pyogenic liver abscess	None	WD	WD	WD	WD	WD	DE	WD	WD	DE

Choi 2014 [17]	*Serratia liquefaciens *complex and *Staphylococcus intermedius* (1)	Cervical necrotizing fasciitis	None	WD	WD	ND	WD	WD	WD	WD	WD	WD

Ha et al. 1999 [30]	*Bifidobacterium longum* (1)	Sepsis	None	WD	WD	ND	WD	WD	WD	WD	DE	ND

Ha and Kim 2003 [26]	No organism growth (1)	Chronic inflammatory epidural granuloma	None	WD	WD	DE	WD	WD	DE	WD	ND	DE

Han et al. 2012 [18]	*Staphylococcus aureus* (1)	Pericardial abscess; sepsis	None	DE	WD	DE	WD	WD	WD	WD	DE	ND

Kang et al. 2012 [14]	*Streptococcus pneumonia* (1)	Psoas abscess; diabetic foot ulcer	None	WD	WD	WD	WD	WD	WD	WD	WD	DE

Jung et al. 2011 [32]	Herpes simplex virus (1)	Skin infection	None	WD	WD	DE	WD	ND	WD	WD	DE	WD

Jung et al. 2014 [22]	*Mycobacterium massiliense* (1)	Localized cutaneous infection	None	WD	WD	ND	WD	WD	DE	WD	DE	DE

Kang and Jeong 2006 [15]	*Streptococcus pyogenes* (1)	Necrotizing fasciitis	None	WD	WD	WD	WD	WD	WD	WD	DE	ND

Kim et al. 2003 [35]	*Spirochetes* (1)	Secondary syphilis	None	WD	WD	WD	WD	WD	DE	WD	WD	ND

Kim et al. 2010 [23]	*Mycobacterium tuberculosis* (3)	Primary inoculation tuberculosis	None	WD	DE	DE	WD	WD	WD	WD	ND	DE

J. W. Kim and Y. S. Kim 2010 [27]	Unidentified (1)	Psoas abscess	None	WD	WD	WD	WD	WD	WD	WD	DE	ND

Kim et al. 2015 [29]	*Actinomyces* species (1)	Abdominal wall actinomycosis	None	WD	ND	ND	WD	WD	DE	WD	WD	WD

Lee et al. 1994 [24]	*Mycobacterium fortuitum* (1)	Cutaneous mycobacterial infection and abscesses	None	WD	WD	ND	WD	WD	DE	WD	WD	DE

Lee et al. 2008 [19]	*Escherichia coli*; MRSA (1)	Necrotizing aortitis	None	WD	ND	DE	WD	WD	DE	WD	ND	DE

Lee et al. 2012 [28]	Unidentified (1)	Cervical epidural abscess	Included	WD	WD	ND	WD	WD	WD	WD	WD	WD

Lim et al. 2013 [34]	Non-O1, Non-O139 *Vibrio cholera* (1)	Septicemia	None	WD	WD	WD	WD	WD	WD	WD	DE	ND

Song et al. 2006 [16]	*Streptococcus pyogenes* (1)	Necrotizing fasciitis	None	WD	WD	WD	WD	WD	DE	WD	DE	DE

Yu et al. 2013 [20]	*Staphylococcus aureus* (1)	Multiple epidural abscess	None	DE	ND	ND	WD	ND	WD	DE	ND	DE

AEs: adverse events, MRSA: methicillin resistant *Staphylococcus aureus*, WD: well documented, DE: documented but not enough for the judgment, ND: not documented, and NA: not applicable; ^*∗*^inclusion of acupuncture specialist among the authors was assessed based on the author's affiliation.

**Table 2:** Author's conclusion in the included studies and causality assessment based on the WHO-UMC criteria.

Study ID (author, yr)	Author's conclusion (Quotation from reports)	Causality assessment^*∗*^
Bang and Lim 2006 [25]	*“Paraplegia might result from complications of an acupuncture therapy.”*	Probable

Cho et al. 2003 [33]	*“We report a case of serious infectious complication caused by acupuncture.”*	Conditional

Cho et al. 2010 [21]	*“We report a case of 59-year-old Korean woman who developed M. abscessus cutaneous infection after multiple acupunctures.”*	Unlikely

Cho et al. 2015 [31]	*“It is plausible that the infection was caused by acupuncture therapy rather than a hematogenous infection.”*	Unlikely

Choi et al. 2013 [13]	*“In this case, we assume that the patient had acupuncture needles and bacteremia after being treated with contaminated was maybe seeded in the liver.”*	Unlikely

Choi 2014 [17]	*“In conclusion, acupuncture and herbal injection should be performed using clean care practices, and NF must be considered as a possible complication in high-risk patients and even also in healthy patients”*	Possible

Ha et al. 1999 [30]	*“Since there were no obvious predisposing conditions preceding anaerobic infection in the young male patient other than acupuncture therapy, it is speculated that the organism was introduced to the blood circulation either from improperly sterilized acupuncture needles or from the colon via minute perforations caused by those needles.”*	Possible

Ha and Kim 2003 [26]	*“…we hypothesize that the epidural granuloma probably formed as the result of focal hemorrhage and low-grade infection by a microorganism after acupuncture…”*	Unassessable

Han et al. 2012 [18]	*“…based on the multifocal acupuncture therapy history of this patient and the absence of previous pericardial disease, the pericardial abscess may have been caused by hematogenous spread of Staphylococcus aureus from the soft tissue infection of the knees.”*	Unlikely

Kang et al. 2012 [14]	*“Based on the aforementioned observations, it was likely that the abscess and septic arthritis were due to acupuncture and moxibustion.”*	Conditional

Jung et al. 2011 [32]	*“We theorize that our patient acquired cutaneous herpes from direct viral inoculation via a contaminated acupuncture needle or reactivation of a cutaneous herpes viral infection due to mechanical trauma.”*	Probable

Jung et al. 2014 [22]	*“We present a case of a localized cutaneous infection due to M. massiliense of the sole associated with acupuncture.”*	Conditional

Kang and Jeong 2006 [15]	*“We present this extremely unusual case of a patient after taking acupuncture who survived severe necrotizing fasciitis of the chest wall following wide debridement of the necrotic tissue and broad-spectrum antibiotic therapy.”*	Unassessable

Kim et al. 2003 [35]	*“We herein report a rare case of secondary syphilis with clinical features of annular pustular psoriasis following the repeated acupuncture and venous drainage by a her doctor.”*	Possible

Kim et al. 2010 [23]	*“Herein, we report 3 cases of primary inoculation tuberculosis resulting from illegal acupuncture in the same nursing home on the same day by a person with no medical training or license.”*	Probable

J. W. Kim and Y. S. Kim 2010 [27]	*“Here, we report the first documented case of psoas abscess caused by acupuncture procedure in a hemodialysis patient.”*	Possible

Kim et al. 2015 [29]	*“Herein, we report an unusual case of abdominal wall actinomycosis which developed in a patient after acupuncture and presented as abdominal wall mass that was first mistaken for abdominal wall invasion of diverticulum perforation.”*	Possible

Lee et al. 1994 [24]	*“In our case, the contaminated acupuncture needle could be an infection source of cutaneous lesions."*	Probable

Lee et al. 2008 [19]	*“In the present case, a long acupuncture needle penetrated from the patient's back is the most suspicious cause for the aortic infection.”*	Unlikely

Lee et al. 2012 [28]	*“In this case, we suspect that wet cupping and/or acupuncture in poorly controlled hygiene might have led to the cervical epidural abscess.”*	Certain

Lim et al. 2013 [34]	*“We report a 56-year-old cirrhotic patient of non-O1, non-O139 septicemia caused by cellulitis of both lower extremities after acupuncture.”*	Unlikely

Song et al. 2006 [16]	*“We report a rare case of necrotizing fasciitis on the face of a 62-year-old man, who had uncontrolled diabetes mellitus following acupuncture treatment.”*	Unassessable

Yu et al. 2013 [20]	*“Multiple epidural abscess after acupuncture.”*	Possible

^*∗*^Causality was assessed according to the WHO-UMC criteria based on the information from the reports on AEs (or complications).

**Table 3:** Reporting status on the specific features of acupuncture treatments in the included studies.

Study ID (author, yr)	Details of acupuncture practice								
	Practitioner's type	Needling site (acupuncture points)	Usage of disposable, sterile needles	Depth of insertion	Needle type	Stimulation method	Acupuncture settings	Disinfection procedure	Appraisal for the appropriateness of acupuncture^*∗*^
Bang and Lim 2006 [25]	ND	DE	ND	DE	DE	ND	ND	ND	Unclear
Cho et al. 2003 [33]	ND	ND	ND	ND	ND	ND	ND	ND	Unclear
Cho et al. 2010 [21]	ND	ND	ND	ND	ND	ND	ND	ND	Unclear
Cho et al. 2015 [31]	DE	ND	ND	ND	ND	ND	WD	ND	Unclear
Choi et al. 2013 [13]	WD	DE	ND	ND	DE	ND	WD	ND	Unclear
Choi 2014 [17]	ND	DE	ND	ND	ND	ND	ND	ND	Unclear
Ha et al. 1999 [30]	ND	DE	ND	ND	DE	ND	DE	ND	Unclear
Ha and Kim 2003 [26]	ND	DE	ND	ND	ND	ND	DE	ND	Unclear
Han et al. 2012 [18]	ND	DE	ND	ND	ND	ND	ND	ND	Unclear
Kang et al. 2012 [14]	ND	DE	ND	ND	ND	ND	ND	ND	Unclear
Jung et al. 2011 [32]	ND	ND	ND	ND	ND	ND	ND	ND	Unclear
Jung et al. 2014 [22]	ND	DE	ND	ND	ND	ND	ND	ND	Unclear
Kang and Jeong 2006 [15]	ND	DE	ND	ND	ND	ND	WD	DE	Unclear
Kim et al. 2003 [35]	ND	DE	ND	ND	ND	ND	WD	ND	Unclear
Kim et al. 2010 [23]	WD	DE	ND	ND	ND	ND	DE	ND	Inappropriate
J. W. Kim and Y. S. Kim 2010 [27]	ND	DE	ND	ND	ND	ND	ND	ND	Unclear
Kim et al. 2015 [29]	ND	DE	ND	ND	ND	ND	ND	ND	Unclear
Lee et al. 1994 [24]	ND	DE	ND	ND	ND	ND	ND	ND	Unclear
Lee et al. 2008 [19]	ND	DE	ND	ND	DE	ND	ND	ND	Unclear
Lee et al. 2012 [28]	WD	WD	ND	WD	ND	ND	WD	DE	Unclear
Lim et al. 2013 [34]	ND	DE	ND	ND	ND	ND	ND	ND	Unclear
Song et al. 2006 [16]	ND	DE	ND	ND	ND	ND	WD	ND	Unclear
Yu et al. 2013 [20]	ND	DE	ND	ND	ND	ND	ND	ND	Unclear

WD: well documented, DE: documented but not enough, ND: not documented, and NA: not applicable; ^*∗*^acupuncture treatment was appraised to be Appropriate when all the acupuncture procedures could not be a probable cause of the adverse events or complications, Inappropriate when any of procedures might be the possible cause of the adverse events or complications, and Unclear when there is not enough information for deciding the appropriateness of acupuncture treatment.

**Table 4:** Recommendation for reporting cases of acupuncture-related infections.

Items	Content
Title	Types of acupuncture practice and AEs (or complications) should be included in the title.

Authors	Acupuncture specialists need to be included among the authors.

Description for the patient	
Demographic data	Sex, age, ethnicity, and residence need to be described.
Preceding conditions or reasons for seeking acupuncture	The patient's diseases or symptoms for seeking acupuncture treatment should be described for assessing appropriateness of acupuncture.
Description on the risk factors for AEs (or complications)	Patient's underlying conditions or cointerventions which might be related to AEs (or complications) need to be declared.

Details of acupuncture intervention [37]	
Acupuncture practitioner's type	Certification, education status, and clinical experience level need to be declared.
Needling sites (acupuncture points)	Location and number of points for acupuncture or needling need to be described in detail using WHO standard acupuncture point locations guideline [38].
Usage of disposable, sterile needles	Usage of disposable, sterile needles should be assessed and reported.
Depth of insertion	Depth and direction of needle insertion should be suggested.
Needle type	Length, diameter, material, and manufacturer of acupuncture needles should be declared.
Stimulation method	Stimulation method for acupuncture including manual, electric stimulation, or other stimulating methods needs to be reported.
Acupuncture settings	Medical institutions or conditions of the physician's office need to be suggested.
Disinfection procedure	Detailed disinfection measure before and after acupuncture should be reported in detail.

Description for the AEs (or complications)	
Time relation between acupuncture and AEs (or complications)	Time line of acupuncture treatment and the occurrence of AE (or complication) symptoms should be suggested clearly.
Explanation on the association between needling site and affected lesion	Relationship between needling site and affected lesion should be evaluated appropriately.
Features of AEs (or complications)	Information on the clinical presentation of AEs (or complications) needs to be suggested sufficiently to assess the causality between acupuncture and the event.
Laboratory or pathological findings	Laboratory or pathological findings related to the AEs (or complications) should be suggested.
Consideration of the other possible causes of AEs (or complications)	Based on the preceding risk factors, other treatments, assessment of acupuncture appropriateness, and other possible causes of AEs (complications) should be evaluated fairly and scientifically.
Appraisal for the appropriateness of acupuncture	Appropriateness of acupuncture practice appraised based on the information about acupuncture intervention, procedure, settings, and disinfection method should be reported.
Causality assessment	Causality category according to the WHO-UMC criteria needs to be suggested based on the clear reason for the decision [39].

Discussion and conclusion	
Previous evidence on the AEs (or complications) related to acupuncture	Previous case reports or literature with rigorous evidence on the current AEs (or complications) needs to be reported.
Conclusion	Conclusion should be written based on the results of the appraisal for the appropriateness of acupuncture practice and causality between acupuncture and the event in a neutral position.
Clinical implication	Preventive measures against current acupuncture-related infection need to be suggested based on the analysis of appropriateness of acupuncture for future safe practice.
